# Supplementary figures and images for: Heavy grazing reduces soil bacterial diversity by increasing soil pH in a semi-arid steppe
Source: PeerJ. 2024 Mar 7;12:e17031. doi: 10.7717/peerj.17031 (PMC10924786; doi:10.7717/peerj.17031)

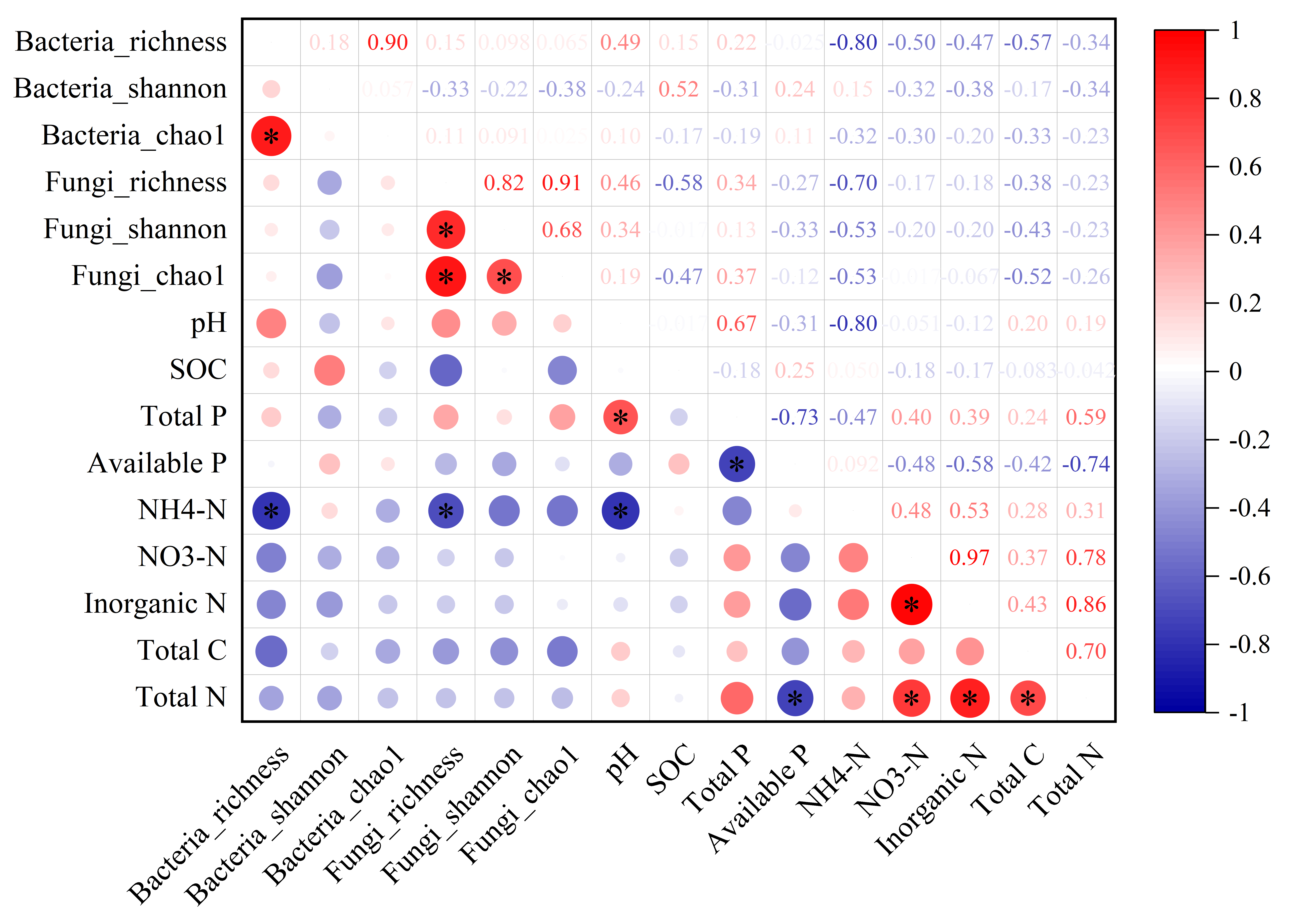

Supplement: Figure S1 — Red and blue denote positive and negative correlations, respectively (* P < 0.05; ** P < 0.01). [file peerj-12-17031-s001.png]

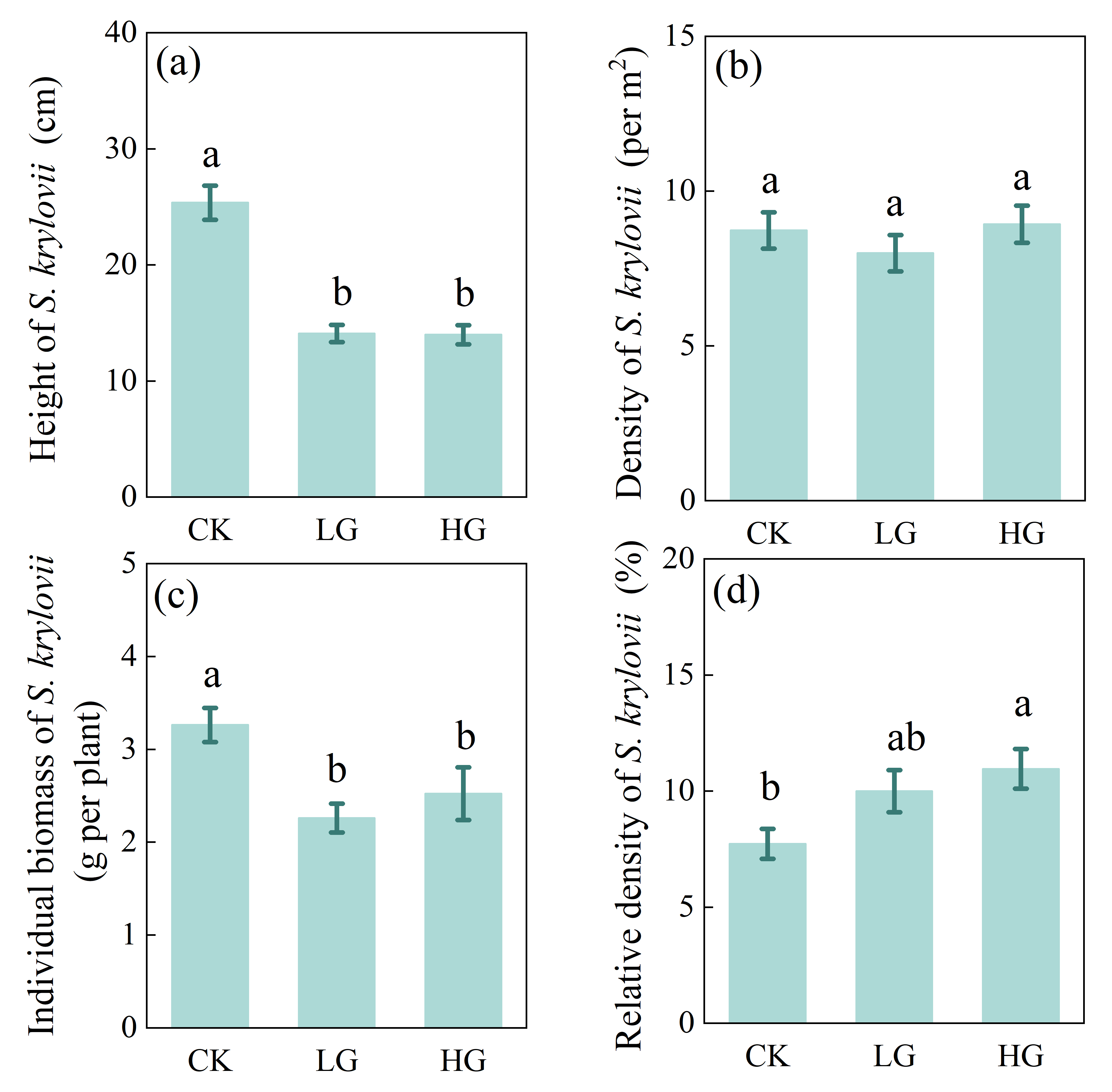

Supplement: Figure S2 — (a)Height of Stipa krylovii, (b) Density of S. krylovii, (c)Individual biomass of S. krylovii, (d) Relative density of S. krylovii. Error bars represent s.e.m. Different lowercase letters indicate significant differences (P < 0.05) among different grazing treatments (i.e., CK, LG or HG treatment). CK: fenced with no grazing, LG: light grazing, HG: heavy grazing. [file peerj-12-17031-s002.png]

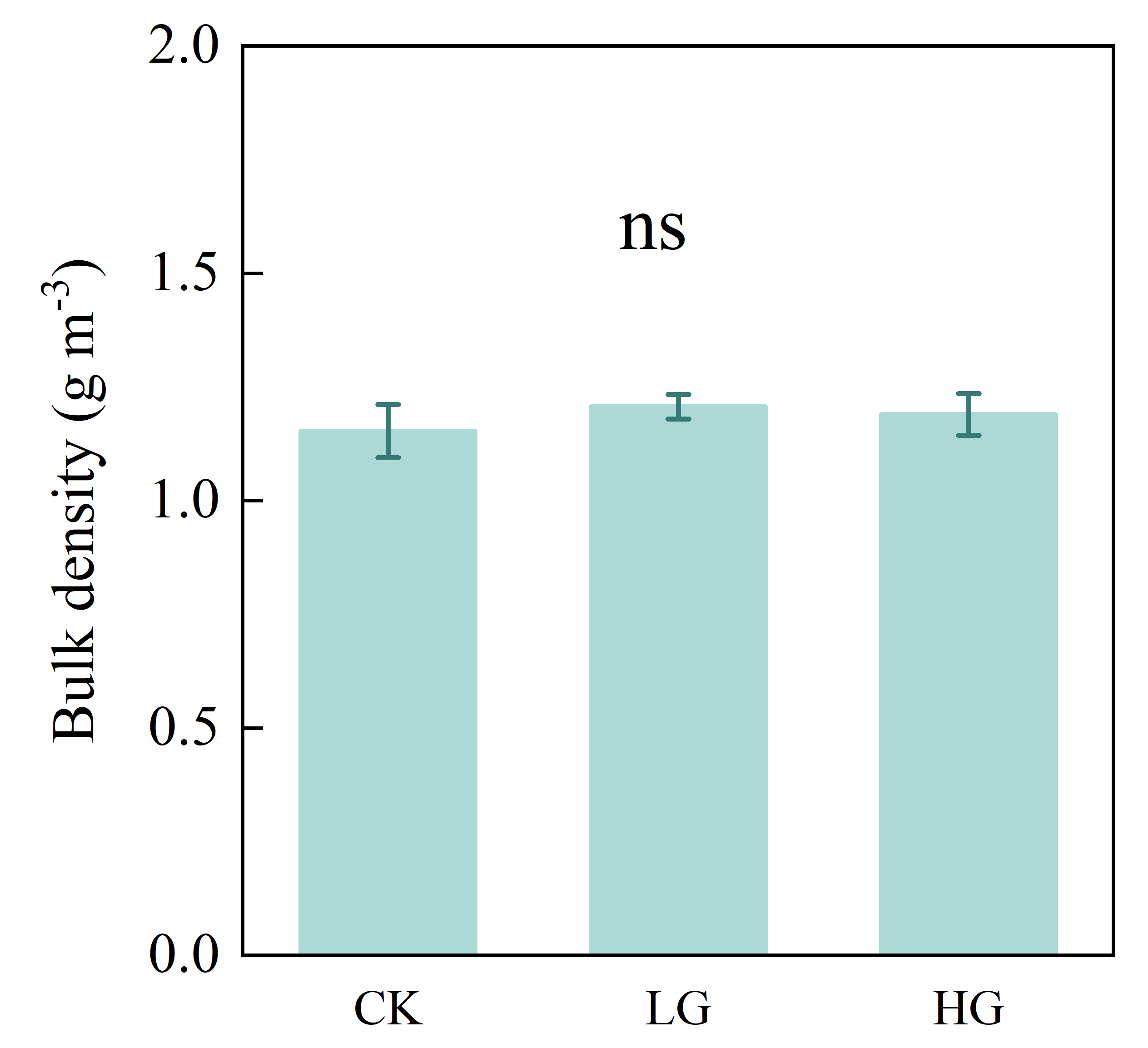

Supplement: Figure S3 — Error bars represent s.e.m. Significance level: non-significance (ns) for P > 0.05. CK: fenced with no grazing, LG: light grazing, HG: heavy grazing. [file peerj-12-17031-s003.png]
